# Structure and Functional Analysis of Promoters from Two Liver Isoforms of CPT I in Grass Carp *Ctenopharyngodon idella*

**DOI:** 10.3390/ijms18112405

**Published:** 2017-11-13

**Authors:** Yi-Huan Xu, Zhi Luo, Kun Wu, Yao-Fang Fan, Wen-Jing You, Li-Han Zhang

**Affiliations:** 1 Key Laboratory of Freshwater Animal Breeding, Ministry of Agriculture, Fishery College, Huazhong Agricultural University, Wuhan 430070, China; xuyihuan1989@163.com (Y.-H.X.); wk901012@126.com (K.W.); fanyaofang@mail.dlut.edu.cn (Y.-F.F.); ywj0861@126.com (W.-J.Y.); zhlh8175@126.com (L.-H.Z.); 2 Collaborative Innovation Center for Efficient and Health Production of Fisheries, Hunan University of Arts and Science, Changde 415000, China

**Keywords:** *Ctenopharyngodon idella*, carnitine palmitoyltransferase I, promoters, peroxisome proliferator-activated receptor, transcriptional regulation

## Abstract

Carnitine palmitoyltransferase I (CPT I) is a key enzyme involved in the regulation of lipid metabolism and fatty acid β-oxidation. To understand the transcriptional mechanism of *CPT Iα1b* and *CPT Iα2a* genes, we cloned the 2695-bp and 2631-bp regions of *CPT Iα1b* and *CPT Iα2a* promoters of grass carp (*Ctenopharyngodon idella*), respectively, and explored the structure and functional characteristics of these promoters. *CPT Iα1b* had two transcription start sites (TSSs), while *CPT Iα2a* had only one TSS. DNase I foot printing showed that the *CPT Iα1b* promoter was AT-rich and TATA-less, and mediated basal transcription through an initiator (INR)-independent mechanism. Bioinformatics analysis indicated that specificity protein 1 (Sp1) and nuclear factor Y (NF-Y) played potential important roles in driving basal expression of *CPT Iα2a* gene. In HepG2 and HEK293 cells, progressive deletion analysis indicated that several regions contained cis-elements controlling the transcription of the *CPT Iα1b* and *CPT Iα2a* genes. Moreover, some transcription factors, such as thyroid hormone receptor (TR), hepatocyte nuclear factor 4 (HNF4) and peroxisome proliferator-activated receptor (PPAR) family, were all identified on the *CPT Iα1b* and *CPT Iα2a* promoters. The TRα binding sites were only identified on *CPT Iα1b* promoter, while TRβ binding sites were only identified on *CPT Iα2a* promoter, suggesting that the transcription of *CPT Iα1b* and *CPT Iα2a* was regulated by a different mechanism. Site-mutation and electrophoretic mobility-shift assay (EMSA) revealed that fenofibrate-induced PPARα activation did not bind with predicted PPARα binding sites of *CPT I* promoters. Additionally, PPARα was not the only member of PPAR family regulating *CPT I* expression, and PPARγ also regulated the *CPT I* expression. All of these results provided new insights into the mechanisms for transcriptional regulation of *CPT I* genes in fish.

## 1. Introduction

Lipids are the major sources of metabolic energy in fish [1]. Body lipid composition results from the balance among deposition of dietary lipids, de novo synthesis of fatty acids and oxidation of fatty acids. While the relations between food intake and lipid deposition as well as nutritional control of fatty acid synthesis are well documented [1], fatty acid catabolism has received little attention. The β-oxidation of fatty acids plays a critical role in the production of energy, and most oxidation occurs in the mitochondria [2]. Carnitine palmitoyltransferase I (EC.2.3.1.21; CPT I), located in outer membranes of mitochondria, controls the flux through β-oxidation and is the main regulatory enzyme of fatty acid oxidation [3,4]. The studies about the structure and transcriptional regulation of *CPT I* gene are useful for the understanding of the β-oxidation in fish. In mammals, three *CPT I* isoforms encoded by distinct genes have been discovered: a liver isoform (*CPT Iα*) [5], a muscle isoform (*CPT Iβ)* [6], and a brain isoform (*CPT Ic*) [7]. In fish, however, due to fish-specific genomic duplication event, various *CPT I* isoforms have been cloned. For example, three α-copies and one β-copy of CPT I was obtained in yellow catfish *Pelteobagrus fulvidraco* [8] and seven complete *CPT I* cDNA sequences (*CPT Iα1a-1a*, *CPT Iα1a-1b*, *CPT Iα1a-1c*, *CPT Iα1a-2*, *CPT I α2a*, *CPT Iα2b1a*, *CPT Iβ*) and a partial cDNA sequence (*CPT Iα2b1b*) were cloned in goby *Synechogobius hasta* [9]. In grass carp, the complete cDNA sequences of three *CPT Iα* genes (*CPT Iα1a*, *CPT Iα1b* and *CPT Iα2a*) and one *CPT Iβ* gene isoforms have successfully been cloned [10,11]. Although these isoforms of CPT I gene can express CPT I protein which catalyzes the same reaction, they have different properties [8]. For example, McGarry and Brown [12] pointed out that mammalian *CPT Iβ* had a much lower IC50 and higher Km for carnitine than *CPT Iα* (from [8]). Lineage- and species-specific genome duplication events can lead to increased diversity in protein regulation and function. At present, while the characteristics of *CPT I* gene and structure prediction as well as its enzyme kinetics are well documented in fish [8,9,11,13,14], mechanisms involving the transcriptional regulations of *CPT I* gene received no attention.

Considering the importance of CPT I in regulating fatty acid oxidation, it is very important and meaningful to explore the regulatory mechanism of *CPT I* mRNA expression. At present, most studies on the mRNA expression and/or activity of *CPT I* isoforms in fish involve the response to either dietary or hormonal treatments [15,16,17,18,19]. However, expression of eukaryotic genes is controlled at the level of transcription initiation. Promoters, which contain cis-acting sequences bound by a wide variety of regulatory factors, control the expression of individual genes. Therefore, it is very important to analyze the structure and function of *CPT I* promoter, which helps to understand the regulatory mechanism of CPT I itself. At present, the promoter of the *CPT Iα* gene has been obtained only in mammals [20,21], but not in fish. The present study hypothesizes that significant differences exist in structure and function of *CPT I* promoters between fish and mammals.

Lipid metabolism is closely controlled by diverse regulatory systems involving many transcription factors. Peroxisome proliferator-activated receptors (PPARs), which belong to ligand-dependent transcription factors, regulate the expression of various genes involved in lipid metabolism [22,23]. Among the PPAR family member, PPARα plays crucial roles in the catabolism of fatty acids by increasing the expression of key lipolytic enzymes (also CPT I) [24,25]. Studies demonstrated that the *PPARα* mRNA expression was positively correlated to *CPT I* mRNA expression [14,26]. Further investigation indicated that PPARα stimulated through a peroxisome proliferator-responsive element (PPRE) in the first and second intron of the human and rat *CPT Iα* genes, respectively [27,28]. PPARγ, involved in the regulation of lipogenesis and lipid storage, preferentially control the transcription of genes in triglyceride synthesis [29]. In an earlier study, Chen et al. [30] found that mRNA expression of *PPARγ* was positively correlated with *CPT I* expression, suggesting a potential regulation of PPARγ on *CPT I* expression. At present, although several evidences suggested that *CPT Iα* was a target gene for PPAR [27,28,29], a lack of knowledge regarding the DNA sequence responsible for this predicted regulatory mechanism has left this a controversial issue. Thus, considering the importance of PPARs in lipid metabolism, it is very important to explore the regulation of CPT I expression by PPARs.

Grass carp (*Ctenopharyngodon idella*) was an important herbivorous freshwater fish widely farmed all over the world because of its good taste and high market price. Its aquaculture yield amounted to 6 million metric tons in China in 2016. In some countries of European and Northern America, grass carp were used to control aquatic plants because of their aggressive feeding on vegetation [31]. At present, grass carp is considered a good model for the study of lipid metabolism because it stores excess fat in liver and adipose tissues under intensive aquaculture. Recently, the draft genome of the grass carp has been released, which is considered a convenient tool for identifying genomic structure of genes involved in lipid metabolism [32]. In the present study, we characterized *CPT Iα1b* and *CPT Iα2a* promoters in grass carp. Their transcriptional regulation by peroxisome proliferators was also explored. These studies will provide new insights into the transcriptional regulatory mechanism of *CPT I* genes in fish.

## 2. Results

Studies indicated that, compared with other isoforms, mRNA levels of *CPT Iα1b* and *CPT 1α2a* were predominant in the liver [10,11]. Therefore, *CPT Iα1b* and *CPT 1α2a* were considered as liver isoforms. To investigate their transcriptional regulatory mechanism, for the first time, we cloned the sequences of promoters of the two liver isoforms (*CPT Iα1b* and *CPT Iα2a*), and explored their functional characteristics in fish.

### 2.1. Identification of Transcription Start Site (TSS)

**Figure 1:**
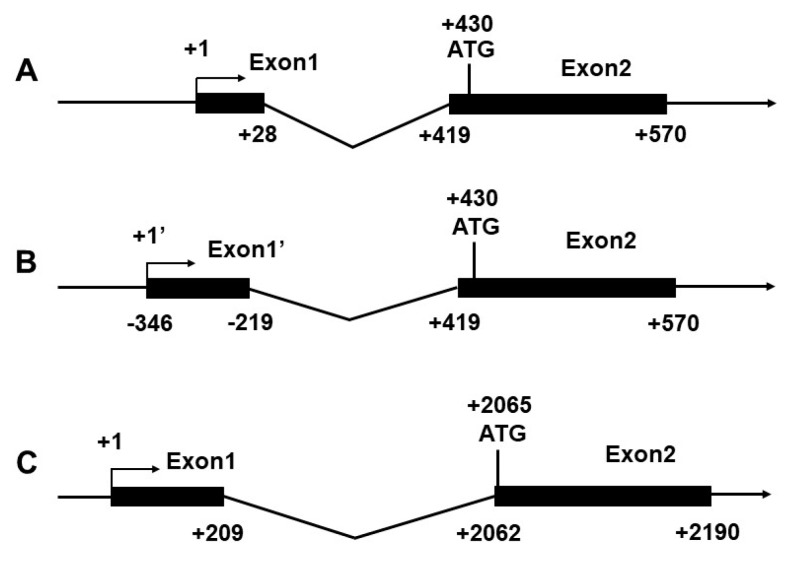
A map of the first two exons in the *CPT Iα1b* and *CPT Iα2a* genes was shown. Exons were denoted by *black rectangles*, introns by a *fold line* and transcriptional direction (5′-3′) by *an arrow line.* The initiation codon (ATG) in exon 2 represented the start site of protein translation. Numbers were relative to the distance from transcription start site (+1). (**A**) structure of transcription start site (TSS) of *CPT Iα1b* gene (**B**) structure of alternative splicing transcription start site (TSS’) of *CPT Iα1b* gene (**C**) structure of transcription start site of *CPT Iα2a* gene.

In the present study, the 2695 bp of *CPT Iα1b* promoter and 2631 bp of *CPT Iα2a* promoter were cloned and submitted to an online transcription factor database (MatInspector) for sequence analysis. RNA ligase-mediated rapid amplification of 5′ cDNA ends (RLM-5′RACE) was performed to identify the TSS of *CPT Iα1b* and *CPT Iα2a* promoters. This amplification generated two different TSSs of *CPT Iα1b* which approximately corresponded to the alternative 5′ splice variants of *CPT Iα1b* mRNA, and one TSS of *CPT Iα2a* without alternative 5′ variant. The first nucleotide of the *CPT Iα1b* gene, mapped to the most upstream position from the grass carp liver cDNA library, was arbitrarily designated as +1′ and the alternative 5′ splicing site was designated as +1 (Figure 1A,B). The first nucleotide of the *CPT Iα2a* gene was designated as +1 (Figure 1C).

### 2.2. DNase I Foot Printing Assay of Core Promoter of CPT Iα1b

**Figure 2:**
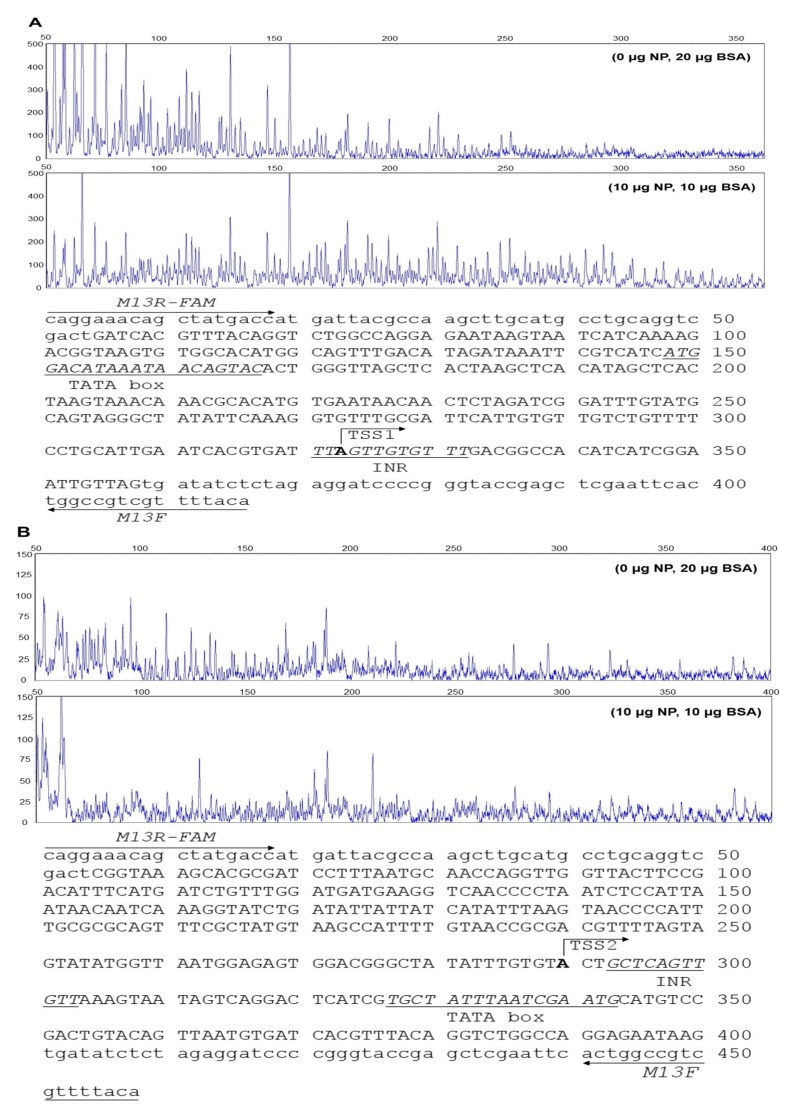
DNase I foot printing assay of proximal promoter of *CPT Iα1b*. (**A**) 303-bp proximal promoter region of *CPT Iα1b* (**B**) 346-bp proximal promoter region of *CPT Iα1b*. The sequence used for FAM-labeled probe was presented, based on the result of DNase I foot printing. Putative binding sequence was underlined and italicized with labels. *Capital letters* indicate the coding sequence of proximal promoter region of *CPT Iα1b*, and *lowcase letters* indicate the partial sequence of pMD-19T vector. The primer sequences used for DNase I foot printing assay M13F and M13R-FAM were labeled by arrows.

Figure 2A showed the core region of *CPT Iα1b* promoter from −268 bp to +37 bp containing transcription start site (TSS1). Predicted TATA-box was located between 148 bp and 167 bp of the FAM-labeled fragment, and the electropherograms around this region presented similar peak patterns between control group (0 µg nuclear proteins, 20 µg bovine serum albumin, BSA) and DNase I digested group (10 µg nuclear proteins, 10 µg BSA). In contrast, the region between 290 bp and 360 bp presented different peak patterns between control group and DNase I digested group, where the initiator (INR) was located. Figure 2B showed the core region of *CPT Iα1b* promoter from −581 bp to −236 bp containing alternative transcription start site (TSS2). Predicted TATA-box on this fragment was located between 327 bp and 343 bp of the FAM-labeled fragment, and the electropherograms on this region were similar between control group (0 µg nuclear proteins, 20 µg BSA) and DNase I digested group (10 µg nuclear proteins, 10 µg BSA). In contrast, the different peak patterns were discovered at the region between 285 bp and 310 bp, where the INR was located. Taken together, these indicated that the INR on the promoter was sufficient for the transcription initiation of *CPT Iα1b* gene.

### 2.3. Sequence Analysis of the CPT Iα1b and CPT Iα2a Promoters

**Figure 3:**
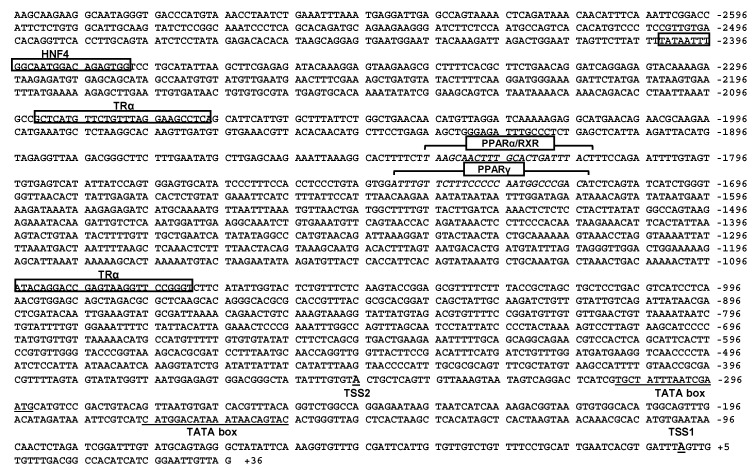
Nucleotide sequence of grass carp *CPT Iα1b* promoter. +1 denoted the transcription start site (*TSS1*) obtained from RLM-5′RACE experiment. *TSS2* presented another transcription start site (−346, TSS′). Numbers indicated the distance from TSS1. The highlighted sequences show putative transcription factor binding sites.

**Figure 4:**
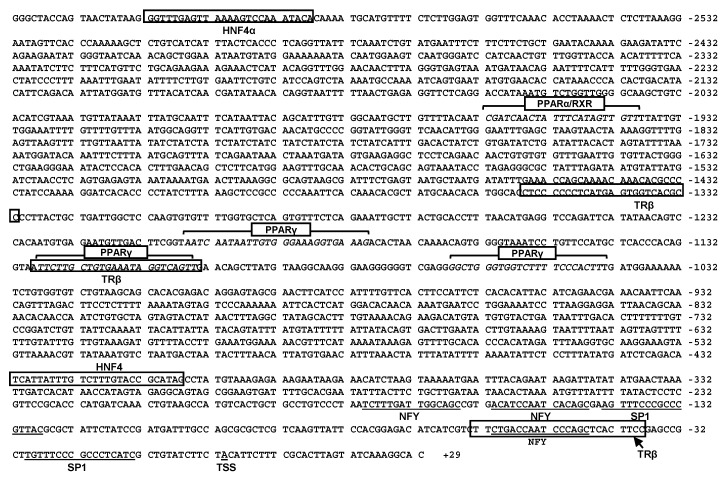
Nucleotide sequence of grass carp *CPT Iα2a* promoter. +1 denotes the TSS obtained from RLM-5′RACE experiment. *Numbers* present distance from TSS. The highlighted sequences show putative transcription factor binding sites.

Several putative core promoter elements close to the TSS on the *CPT Iα1b* promoter, including two TATA-box (TBP) located from −160 bp to −176 bp and from −293 bp to −309 bp, and two initiator (INR) located at −2 bp to +10 bp (TSS1) and −333 bp to −343 bp (TSS2), were identified (Figure 3). Meanwhile, on the core region of *CPT Iα2a* promoter, three CCAAT-box (NF-Y) were identified, located at −46 bp to −60 bp, −146 bp to −160 bp and −165 bp to −179 bp, respectively. Besides, two GGGCGG-box (Sp1), located at −13 bp to −29 bp and −127 bp to −143 bp, were also identified on the core promoter of *CPT Iα2a* (Figure 4). Some relevant TFBSs of *CPT Iα1b* and *CPT Iα2a* were presented in Figure 3 and Figure 4. There were two thyroid hormone receptor α (TRα) binding sites on the *CPT Iα1b* promoter at the position −1070 bp to −1094 bp and −2067 bp to −2091 bp, and three thyroid hormone receptor β (TRβ) binding sites on the *CPT Iα2a* promoter, at the position −39 bp to −63 bp, −1103 bp to −1127 bp and −1331 bp to −1355 bp, respectively. In addition, we discovered one HNF4 binding site on the *CPT Iα1b* promoter, located at −2379 bp to −2403 bp, one HNF4 binding site on the *CPT Iα2a* promoter, located at the position −406 bp to −430 bp, and one HNF4α binding site on the *CPT Iα2a* promoter, located at the position −2587 bp to −2611 bp. Moreover, analysis using MatInspector database revealed two PPAR binding sites on the *CPT Iα1b* promoter and four PPAR binding sites on the *CPT Iα2a* promoter. Among these sites, one PPARα/RXR binding site located at the position −1814 bp to −1836 bp and one PPARγ binding site located at the position −1719 bp to −1741 bp were predicted on the *CPT Iα1b* promoter. Meanwhile, there were four important binding sites of transcriptional factors on the *CPT Iα2a* promoter, distributed at the position −1939 bp to −1961 bp (PPARα/RXR binding site), −1179 bp to −1201 bp (PPARγ binding site), −1104 bp to −1136 bp (PPARγ binding site) and −1044 bp to −1066 bp (PPARγ binding site).

### 2.4. Deletion Assay of the CPT Iα1b and CPT Iα2a Promoter

**Figure 5:**
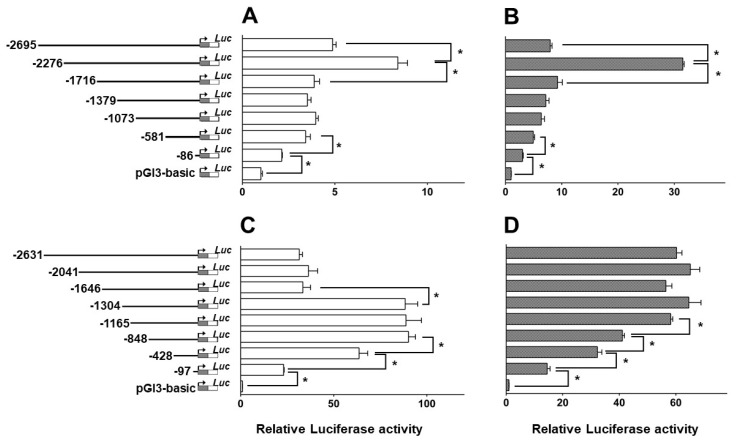
5′ Unidirectional deletion analysis of the *CPT Iα1b* and *CPT Iα2a* promoter regions for grass carp. Schematic diagrams of truncated promoters were shown at the left panel. The corresponding luciferase reporter assay results were shown in the right panel. Promoter activity of constructs is presented with the values of relative light unit. A series of plasmids containing 5′ unidirectional deletions of the *CPT Iα1b* promoter region fused in frame to the luciferase gene were transfected into HepG2 cells (**A**) and HEK293 cells (**B**), and a series of plasmids containing 5′ unidirectional deletions of the *CPT Iα2a* promoter region were transfected into HepG2 cells (**C**) and HEK293 cells (**D**). Values represent the ratio between firefly and renilla luciferase activities, normalized to the control plasmid pGl3-Basic. Results were expressed as the mean ± SEM of three independent experiments (Student’s *t*-test, * *p* < 0.05).

Deletion analysis of *CPT Iα1b* and *CPT Iα2a* promoters was presented in Figure 5. The reporter activity for each serial deletion was compared with the activity of pGl3-basic vector, and the pGl3-basic was chosen as the baseline. Figure 5A showed the result of deletion assay of the *CPT Iα1b* promoter sequence from −2695 bp to −86 bp in HepG2 cells. Deletion of the region from −2276 bp to −2695 bp significantly increased the relative luciferase activity of the promoter. Subsequent deletion to −1716 bp significantly decreased the relative luciferase activity. Deletion of the sequence from −581 bp to −1716 bp showed no significant effect, whereas deletion of the sequence from −581 bp to −86 bp significantly decreased the relative luciferase activity. Figure 5B showed the result of deletion assay of the *CPT Iα1b* promoter in HEK293 cells. Deletion of the sequence from −2695 bp to −2276 bp significantly increased the relative luciferase activity, and the sequence deletion from −2276 bp to −1716 bp significantly reduced the activity. Subsequent deletion to −581 bp presented no significant effects on the relative luciferase activity. The sequence deletion from −581 bp to −86 bp significantly decreased the relative luciferase activity.

Figure 5C presented the result of deletion assay of the *CPT Iα2a* promoter sequence from −2631 bp to −97 bp in HepG2 cells. The relative luciferase activity of *CPT Iα2a* promoter showed no significant difference from −2631 bp to −1646 bp. Deletion of the sequence from −1646 bp to −1304 bp significantly increased the relative luciferase activity. Subsequent deletion to −848 bp presented no significant effects on the relative luciferase activity. Deletion of the sequence of −848 bp to −428 bp and −428 bp to −97 bp significantly decreased the relative luciferase activity. Figure 5D showed the result of deletion assay of *CPT Iα2a* promoter in HEK293 cells. Deletion of the sequence from −2631 bp to −1165 bp presented no significant effects on the relative luciferase activity. All of these sequence deletions from −1165 bp to −848 bp, −848 bp to −428 bp and −428 bp to −97 bp significantly decreased the luciferase activity.

### 2.5. Site-Mutation Analysis of PPAR Binding Sites

**Figure 6:**
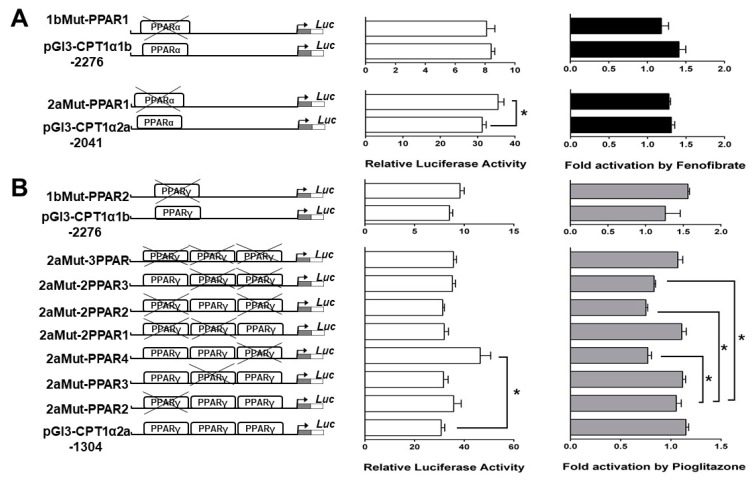
Analysis of putative PPAR binding sites by site-directed mutagenesis. Site-mutation constructs are presented in the left panel. Promoter activity of constructs is presented in the middle. Promoter activity treated with agonist was presented in the right panel. (**A**) site-mutations of PPARα binding sites on pGl3-CPTIα1b-2276 and pGl3-CPTIα2a-2041 vectors (**B**) site-mutation of PPARγ binding sites on pGl3-CPTIα1b-2276 and pGl3-CPTIα2a-1304 vectors. Values represent the ratio between firefly and renilla luciferase activities, normalized to the control plasmid pGL3-Basic. Bars are the mean ± SEM of three independent experiments (Student’s *t*-test, * *p* < 0.05).

Site-mutation analysis was used to evaluate the contribution of each PPAR binding site to the basal expression of the grass carp *CPT Iα1b* and *CPT Iα2a* genes in HepG2 cells (Figure 6). The disruption of the −1814/−1836 PPARα binding site did not change the relative luciferase activity against the wild-type pGl3-CPTIα1b-2276, and disruption of the −1814/−1836 PPARα binding site did not influence the fenofibrate-induced change of luciferase activity, indicating that −1814/−1836 PPARα binding site did not contribute to the transcriptional response of *CPTIα1b* gene to fenofibrate (Figure 6A). The disruption of the −1939/−1961 PPARα binding site significantly up-regulated the relative luciferase activity against the wild-type pGl3-CPTIα2a-2041. In contrast, disruption of the −1939/−1961 PPARα binding site did not influence the fenofibrate-induced change of luciferase activity induced, suggesting that the −1939/−1961 sequence did not contribute to the transcriptional response of *CPTIα2a* gene to fenofibrate. We also disrupted each PPARγ binding site by site-directed mutagenesis in the context of the pGl3-CPTIα1b-2276 and pGl3-CPTIα2a-1304 vectors, respectively; meantime, three double mutants and one triple mutant of PPARγ binding site were produced on the pGl3-CPTIα2a-1304 vector (Figure 6B). The disruption of the −1719/−1741 PPARγ binding site did not change the relative luciferase activity against the wild-type pGl3-CPTIα1b-2276, and disruption of the −1719/−1741 PPARγ binding site did not influence the pioglitazone-induced change of luciferase activity, suggesting that −1719/−1741 PPARγ binding site did not contribute to the transcriptional response of CPTIα1b gene to pioglitazone. Disruptions of the PPARγ binding sites on pGl3-CPTIα2a-1304 vectors showed that the −1044/−1066 PPARγ binding site up-regulated relative luciferase activity against the pGl3-CPTIα2a-1304. Other mutant vectors, including double and triple mutant of PPARγ binding sites, presented no significant difference in luciferase activities against the wild-type pGl3-CPTIα2a-1304, indicating that the −1044/−1066 PPARγ binding site possibly played a negative regulatory role in *CPTIα2a* transcription. In addition, disruption of the −1719/−1741 PPARγ binding site reduced the luciferase activity induced by pioglitazone, and disruption of −1719/−1741 PPARγ binding site along with either −1179/−1201 PPARγ binding site or −1104/−1136 PPARγ binding site also reduced the luciferase activity induced by pioglitazone, suggesting that −1719/−1741 PPARγ binding site contributed to the transcriptional response of *CPTIα2a* to pioglitazone. Taken together, these results indicated that PPARα could not regulate the transcription of *CPT Iα1b* and *CPT Iα2a* at their predicted binding sites, and the transcription of the grass carp *CPT Iα2a* gene expression could be controlled by PPARγ.

### 2.6. EMSA of Each PPAR Binding Sequence

**Figure 7:**
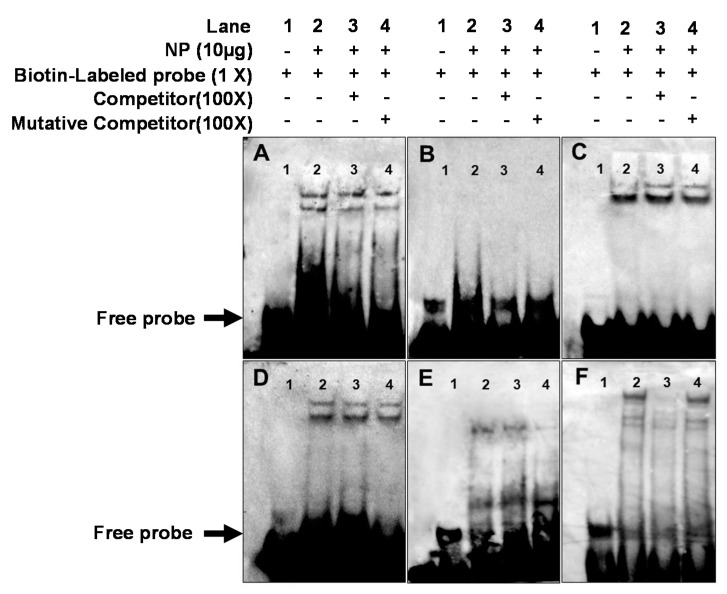
Electrophoretic mobility-shift assay (EMSA) of putative PPAR binding sequences. The 5′-biotin labeled double-stranded oligomers were incubated with HepG2 nuclear extract (*NP*). A 100-fold excess of the competitor and Mutative competitor oligomers was added to the competition and mutant competition assay, respectively. The oligonucleotide sequences are given in Table 1. (**A**) PPARα/RXR binding sequence located at −1814 bp to −1836 bp of CPT Iα1b promoter (**B**) PPARγ binding sequence located at −1719 bp to −1741 bp of *CPT Iα1b* promoter (**C**) PPARα/RXR binding sequence located at −1939 bp to −1961 bp of *CPT Iα2a* promoter (**D**) PPARγ binding sequence located at −1179 bp to −1201 bp of *CPT Iα2a* promoter (**E**) PPARγ binding sequence located at −1104 bp to −1136 bp of *CPT Iα2a* promoter (**F**) PPARγ binding sequence located at −1044 bp to −1066 bp of *CPT Iα2a* promoter.

Having demonstrated that the putative PPAR binding site was important for the transcriptional activities of *CPT Iα1b* and *CPT Iα2a* genes, we next examined whether PPARs could bind to this site directly. We used EMSA assay to confirm this mechanism (Figure 7). Two close weak bands were observed at the −1814/−1836 PPARα binding sequence of *CPT Iα1b* promoter, and neither a 100-fold excess unlabeled probe nor a 100-fold excess unlabeled point-mutated probe could compete out the labeled probe, indicating that this sequence was not bound by PPARα (Figure 7A). Only the free probe band was discovered at the −1719/−1741 PPARγ binding sequence of *CPT Iα1b* promoter (Figure 7B), suggesting that this sequence was not bound by any transcriptional factors. A strong band close to a weak band was observed at the −1939/−1961 PPARα binding sequence of *CPT Iα2a* promoter, and neither a 100-fold excess unlabeled probe nor a 100-fold excess unlabeled point-mutated probe could compete out the labeled probe, indicating that this sequence was not bound by PPARα (Figure 7C). Similarly, Figure 7D and E also indicated that the −1179/−1201 and −1104/−1136 PPARγ binding sequences of *CPT Iα2a* promoter were not bound by PPARγ. Only the sequence corresponding to the −1104/−1066 PPARγ binding site of the *CPT Iα2a* promoter could bind with proteins from HepG2 nuclear extract (NP) and be disrupted by a 100-fold excess of unlabeled wild-type, and restored by a point-mutant probe (Figure 7F), confirming that −1044/−1066 PPARγ binding site of the *CPT Iα2a* promoter could react with PPARγ.

## 3. Discussion

The reaction catalyzed by CPT I is a rate-controlling step in the pathway of LCFA β-oxidation. Currently, five isoforms of *CPT I* genes (*CPT Iα1a*, *CPT Iα1b*, *CPT Iα2a*, *CPT Iα2b* and *CPT Iβ*) were identified in grass carp (*C. idella*) [10,11]. Moreover, these studies indicated that, compared with other isoforms, mRNA levels of *CPT Iα1b* and *CPT 1α2a* were predominant in the liver. Therefore, *CPT Iα1b* and *CPT 1α2a* were considered as the liver isoform. To investigate their transcriptional regulatory mechanism, for the first time, we cloned the sequences of *CPT Iα1b* and *CPT Iα2a* promoters in fish, and explored their functional characteristics.

In the present study, we found two TSSs of *CPT Iα1b* corresponding to the alternative 5′ splice variants of *CPT Iα1b* mRNA. Studies suggested that alternative TSSs usually occurred in the proximal promoter of genes lacking TATA and CCAAT boxes [33]. Batarseh et al. [34] pointed out that multiple TSSs were typically TATA-less and they were located within CpG islands. Park et al. [19] found that the rat L-CPT I (*CPT Iα*) promoter was GC rich and TATA-less and had an alternative transcription initiation. However, our present study found some variations in TSSs of the *CPT Iα1b* promoter in grass carp. Grass carp *CPT Iα1b* promoter was AT-rich and contained two TATA elements without canonical CpG islands, but DNase I foot printing assay showed that both TATA elements were not protected from DNase I digestion, whereas the INR, which encompassed the TSS, was protected from DNase I digestion. These phenomena indicated that the basal transcription of the *CPT Iα1b* gene required the INR to position the basal transcription machinery. In agreement with our study, Smale and Kadonaga [35] pointed out that the INR was located at the TSS and it was independent of, or in synergy with the TATA box. Thus, our results suggested that the basal transcription of the *CPT Iα1b* gene might be mediated through an INR-independent mechanism. For *CPT Iα2a* gene, the present study indicated that the core promoter of grass carp *CPT Iα2a* was GC-rich and did not contain a TATA box.

In agreement with rat *CPT Iα* gene [21], our study indicated that the proximal promoter region of *CPT Iα2a* contained several Sp1 and NF-Y binding sites, whereas only one transcription initiation was identified on the promoter. Steffen et al. [21] pointed out that the Sp1, Sp3 and NF-Y factors played major roles in driving basal expression of rat *CPT Iα* gene. Sp1, a ubiquitously expressed prototypic C2H2-type zinc finger protein, can activate or repress transcription after physiological and pathological stimuli [36,37]. Studies demonstrated that multiple Sp1 binding sites were a common feature of TATA-less promoters [35]. Moreover, the Sp1 can bind GC-rich motifs and regulate the expression of genes via protein-protein interactions or interplay with other transcription factors and/or components of the transcriptional machinery [37]. NF-Y, one of the major transcriptional factors binding to the CCAAT box, may interact with Sp1 to regulate transcription of various genes [38,39]. In agreement with these studies, the present study indicated that Sp1 and NFY factors were identified on the core region of *CPT Iα2a* promoter in a similar manner, indicating a similar transcription initiation for *CPT Iα2a* transcription. Taken together, our study indicated that transcription initiation of the *CPT Iα1b* and *CPT Iα2a* genes presented different mechanisms, suggesting that the expression of two genes from grass carp was induced by different transcriptional initiation.

Identification of TFBSs is very important for deciphering the mechanisms of gene regulation [40]. To better understand the regulation of *CPT Iα1b* and *CPT Iα2a* at the transcriptional level, we functionally characterized the *CPT Iα1b* and *CPT Iα2a* promoters of grass carp. The present study identified a cluster of TFBS, such as TR, HNF4 and PPAR family, on the grass carp *CPT Iα1b* and *CPT Iα2a* promoters. Similarly, Jackson-Hayes et al. [41] showed that the rat *CPT Iα* gene had a thyroid hormone response element (TRE) which was required for the thyroid hormone receptor (TR) binding. In the present study, several TREs were also observed on the grass carp *CPT Iα1b* and *CPT Iα2a* promoters. Interestingly, our study found that *CPT Iα1b* and *CPT Iα2a* promoters were bound by different isoforms of TRs. The two TREs on the *CPT Iα1b* promoter were only for TRα binding, and the three TREs on the *CPT Iα2a* promoter were only for TRβ binding. In fish, TRα and TRβ were expressed at different developmental stages, suggesting their functional differentiation [42]. Additionally, TRα and TRβ were differentially regulated by systemic thyroid status in fish [43]. Thus, these studies strongly suggested that the transcriptions of *CPT Iα1b* and *CPT Iα2a* genes were regulated through different mechanism in the liver.

In the present study, deletion analysis indicated that several regions of the promoters contained a potential cis-active element(s) which enhanced/decreased transcriptional activities of the grass carp *CPT Iα1b* and *CPT Iα2a* genes. Furthermore, the regions of the *CPT Iα1b* and *CPT Iα2a* promoters presented different reporter activities in HepG2 and HEK293 cells. Obviously, the enhancing/decreasing reporter activity indicated the existence of potential positive/negative regulators on the regions, respectively. For the promoter of *CPT Iα1b* gene, we found that, compared to the region between −1716 and 1379 bp, the luciferase activity between the region from −2276 bp to −1716 bp significantly increased in HepG2 and HEK293 cells. Interestingly, we noticed that the −2067/−2091 TRα binding site, −1939/−1961 PPARα binding site and −1719/−1741 PPARγ binding site were located at the region from −2276 bp to −1716 bp, which was reported to correlated with *CPT I* expression in mammals [41,44,45,46]. For the promoter of *CPT Iα2a* gene, we discovered that the luciferase activity increased from the TSS to −848 bp in HepG2 cells, whereas the activity increased from TSS to −1165 bp in HEK293 cells, indicating a different regulation at the region from −848 bp to −1165 bp between the two kinds of cell lines. In the meantime, we found the −1103/−1127 TRβ binding site, −1044/−1066 PPARγ binding site and −1104/−1126 PPARγ binding site were located in this region. Besides, the luciferase activity declined at the region from −1304 bp to −1646 bp in HepG2 cells, but not in HEK293 cells, and this region contained the −1331/−1355 TRβ binding site. Considering different TREs on *CPT Iα1b* and *CPT Iα2a* promoter, TR enhanced the promoter activity of corresponding genes and might play important roles in regulating the expression of CPT Iα2a in different tissues. Moreover, we also discovered that the luciferase activity declined in the region from −2695 bp to −2276 bp of *CPT Iα1b* and the region from −1304 bp to −1646 bp of *CPT Iα2a* promoter in HepG2 cells. Obviously, some negative regulators binding on these regions regulated *CPT Iα1b* and *CPT Iα2a* expression. In addition, studies suggested that the luciferase activity declined in the deletion region from more that −6000 bp to −1653 bp on the promoter of rat *CPT Iα* [21]. However, the present study indicated that the transcription activity increased on the upstream of the promoters of grass carp *CPT Iα1b* and *CPT Iα2a*, suggesting that positive regulators existed on the upstream region of *CPT Iα1b* and *CPT Iα2a* promoters. Thus, it appears that the regulation of CPT Iα1b and CPT Iα2a transcription was more complicated in fish than mammals.

PPARs are key transcriptional factors which mediate the regulation of many enzymes related with lipid metabolism [44]. Studies suggested that *CPT I* was one of the target genes of PPARα [27,45]. In our study, the activities of *CPT Iα1b* and *CPT Iα2a* promoters were induced by fenofibrate, PPARα agonist. However, site-directed mutagenesis and EMSA analysis indicated that *CPT Iα1b* and *CPT Iα2a* were not regulated through those predicted PPARα binding sites. Accordingly, the reporter activities were up-regulated by fenofibrate, indicating that other potential PPARα binding sites or other related factors existed on the promoter. For instance, studies suggested that PPARα-induced activation of *CPT Iα* gene was enhanced in a ligand-dependent manner by PGC-1 [46], and PGC-1, as a co-activator, can activate gene transcription through HNF4α [47]. Additionally, studies established the necessity of the first intron in the transcriptional regulation of the CPT Iα gene [26,40]. Taken together, the induction of *CPT Iα1b* and *CPT Iα2a* by fenofibrate may involve several nuclear factors and/or other promoter regions of the gene.

PPARγ is one of transcriptional factors which plays important roles in lipogenesis. The present study indicated that transcription of grass carp *CPT I* was regulated by PPARγ. Pioglitazone, the agonist of PPARγ [48], could increase the activity of grass carp *CPT Iα2a* promoter, and site-mutation on the −1044/−1066 PPARγ binding site reduced the activity. Moreover, EMSA assay confirmed that the sequence at −1044 bp to −1066 bp was a functional binding locus. Similarly, Gilde et al. [49] found that overexpression of PPARγ in cardiomyocytes was accompanied by basal and ligand-activated transcription of the *CPT I* promoter. Patsouris et al. [50] reported that PPARγ compensated for PPARα by mediating the HFD-induced up-regulation of PPARα target genes involved in fatty acid oxidation in PPARα-null mice. Moreover, studies suggested that the addition of a classical agonist ligand promoted the dissociation of the co-repressor and the binding of co-activator proteins resulting in an enhancement in the basal transcriptional level of specific genes [51,52]. Stanley [53] found that the PPARγ-directed pioglitazone enhanced the affinity for co-activators and decreased the affinity for co-repressor on PPARγ, indicating that PPARγ possibly activated gene transcription by causing the dissociation of co-repressors and promoting the association of co-activator proteins. Similarly, the present study indicated that site-directed mutagenesis on the −1044/−1066 PPARγ binding site possibly decreased the activity of *CPT Iα2a* promoter, and pioglitazone-induced activation of PPARγ could up-regulate the activity of *CPT Iα2a* promoter. These evidences indicated that PPARγ probably played an important role in regulating CPT Iα2a transcription and compensated for PPARα-induced expression of lipolytic genes in fish.

In summary, the 2695-bp *CPT Iα1b* and 2631-bp *CPT Iα2a* promoters in grass carp had been cloned and characterized. The promoters of *CPT Iα1b* and *CPT Iα2a* genes showed the different structures in their core regions. Several putative TFBSs had been predicted in their promoter regions. Analysis of 5′ deletion mutants presented the regulatory characteristics of *CPT Iα1b* and *CPT Iα2a* promoters. Fenofibrate activated the activities of *CPT Iα1b* and *CPT Iα2a* promoters. PPARγ played an important role in regulating *CPT I* expression. The present study provided new insights into the regulatory mechanisms of liver isoforms of *CPT I* genes in fish.

## 4. Materials and Methods

### 4.1. Experimental Animals and Cells

Juvenile grass carp was obtained from Hubei Honghu Fisheries Farm (Jingzhou, Hubei Province, China). HepG2 and HEK293 cell lines were obtained from the Cell Resource Center in Fishery College of Huazhong Agricultural University. We ensured that the experiments were performed in accordance with the experimental protocols of Huazhong Agricultural University (HZAU) and approved by the ethics committee of HZAU (identification code: Fish-2015-0324, Date: 24 March 2015).

### 4.2. Rapid Amplification of 5′ cDNA Ends (5′ RACE)

The TSSs of *CPT Iα1b* and *CPT Iα2a* genes were determined using the GeneRacer Kit (Invitrogen, Carlsbad, CA, USA), according to the manufacturer’s instructions. Briefly, total RNA was isolated from the liver tissue using TRIzol Reagent (Invitrogen), and then the 5′-ready cDNA libraries were prepared using reverse transcription kit (Invitrogen). Nested PCR was performed using a commercial nested 5′ primer (Invitrogen) in combination with a reverse gene-specific primer complementary to *CPT Iα1b* and *CPT Iα2a* genes. The PCR reactions were performed using TaKaRa PrimeSTAR^®^ HS DNA Polymerase kit (TaKaRa, Otsu, Japan) under the following PCR conditions: pre-incubation at 94 °C for 3 min, 30 cycles of 94 °C for 15 s, 60 °C for 30 s and 72 °C for 1 min. Amplified PCR products were gel-purified and subcloned into pMD19-T for sequencing (Tsingke, Wuhan, China).

### 4.3. Cloning of Promoter and Plasmid Construction

Based on the published draft genome of grass carp [32], we cloned the sequences of *CPT Iα1b* and *CPT Iα2a* promoters. Genomic DNA was extracted from grass carp tail fins using a commercial kit (Omega, Norcross, GA, USA). For amplification of the *CPT Iα1b* and *CPT Iα2a* promoter sequences, specific primers with overlapping sequence were designed and listed in Table 1. For the generation of the luciferase reporter constructs, the PCR product and pGl3-Basic vectors (Promega, Madison, WI, USA) were purified and digested using corresponding endonucleases, and then products were ligated using ClonExpress™ II One Step Cloning Kit (Vazyme, Piscataway, NJ, USA). According to the distance from its TSS, the plasmids were named as pGl3-CPTIα1b-2695 and pGl3-CPTIα2a-2632, respectively. Plasmids pGl3-CPTIα1b-2276, pGl3-CPTIα1b-1716, pGl3-CPTIα1b-1073, pGl3-CPTIα1b-581, pGl3-CPTIα1b-86, pGl3-CPTIα2a-2041, pGl3-CPTIα2a-1646, pGl3-CPTIα2a-1304, pGl3-CPTIα2a-1165, pGl3-CPTIα2a-848, pGl3-CPTIα2a-428 and pGl3-CPTIα2a-97, which contained unidirectional deletions of the promoter regions, were generated with the Erase-a-Base system (Promega) using templates of pGl3-CPTIα1b-2695 and pGl3-CPTIα2a-2632, respectively. The PCR reactions were performed using TaKaRa PrimeSTAR^®^ HS DNA Polymerase kit (TaKaRa) as mentioned above. All plasmids were sequenced in a commercial company (Tsingke).

### 4.4. Sequence Analysis

For sequence analysis of promoters of *CPT Iα1b* and *CPT Iα2a* genes in grass carp, putative TFBSs were predicted by MatInspector online (http://www.genomatix.de/). Nucleotide sequences of *CPT Iα1b* and *CPT Iα2a* promoters were compared with DNA sequences present in the GenBank database (http://www.ncbi.nlm.nih.gov/genbank/) and the UCSC Genome Browser (http://genome.ucsc.edu/).

### 4.5. DNase I Foot Printing Assay

DNase I foot printing assays were performed based on the method of Zianni et al. [54]. In brief, 303-bp and 346-bp proximal regions of *CPT Iα1b* promoter, which contained two TSSs (TSS1 and TSS2, respectively), were PCR amplified and cloned into pMD-19T vector (TaKaRa). Then, the amplicons were used as the template for further preparation of fluorescent 6-carboxy-fluorescein (FAM)-labeled probes with M13F and M13R-FAM to label the coding strand. After agarose gel electrophoresis, the FAM-labeled probes were purified by Gel Extraction Kit (Omega, USA) and quantified with NanoDrop 2000 (Thermo, Waltham, MA, USA). 10 µg of proteins extracted from HepG2 cell lines were incubated with 500 ng of probes in the same binding buffer based on Zianni et al. [53]. DNase I digestion was performed for 3 min at room temperature and then terminated by the addition of DNase I stop solution (Promega). Digested samples were precipitated with alcohol and then analyzed with the 3730 DNA Analyzer in the commercial company (Tsingke).

### 4.6. Transfections and Luciferase Assays

HepG2 and HEK293 cells were cultured in DMEM medium supplemented with 10% (*v*/*v*) heat-inactivated FBS (Invitrogen) and 2 mM l-glutamine in a humidified atmosphere with 5% CO_2_ at 37 °C. Prior to the transient transfection, HepG2 or HEK293 cells were seeded in 24-well cell culture plate at a density of 1.2 × 10^5^ and cultured for 24 h to reach 70–80% convergence. Plasmids were transiently transfected into HepG2 or HEK293 cells using Lipofectamine™ 2000 (Invitrogen) following the manufacture’s protocol. All reporter plasmids were used in equimolar amounts in Opti-MEM (Invitrogen), and they were co-transfected with 35 ng pRL-TK as the control. After 4 h, the transfection medium was replaced by 10% FBS-DMEM. Then, with 24-h incubation, cells were harvested to assay the relative luciferase activity by Dual-Luciferase Reporter Assay System (Promega). The relative luciferase activity was presented as the ratio of firefly luciferase to renilla luciferase. Results were normalized to the control reporter pGl3-Basic. All experiments were performed in triplicates and measured at least three times.

### 4.7. Site-Mutation Analysis of PPAR Binding Sites on the Grass Carp CPT Iα1b and CPT Iα2a Promoters

To identify the corresponding PPAR binding sites on the grass carp *CPT Iα1b* and *CPT Iα2a* promoters, we performed site-directed mutagenesis according to the manufacture instruction of QuickChange II Site-Directed Mutagenesis Kit (Vazyme). pGl3-CPTIα1b-2276, pGl3-CPTIα2a-2041 and pGl3-CPTIα2a-1304 were used as templates. The mutagenesis primers were listed in Table 1, and the PCR reactions were performed as mentioned above. These mutant constructs were named as 1bMut-PPAR1, 1bMut-PPAR2, 2aMut-PPAR1, 2aMut-PPAR2, 2aMut-PPAR3, 2aMut-PPAR4, 2aMut-2PPAR1, 2aMut-2PPAR2, 2aMut-2PPAR3 and 2aMut-3PPAR, respectively. Then the constructs and pRL-TK were co-transfected into HepG2 cell lines using Lipofectamine^TM^ 2000 following the manufacture’s protocol. After 4 h, the transfection medium was replaced by 10% FBS-DMEM with 50 μM fenofibrate or 10 μM pioglitazone. After 24-h incubation, cells were harvested to assay the luciferase activity according to the procedure above.

### 4.8. Electrophoretic Mobility-Shift Assay (EMSA)

EMSA was performed to confirm the functional PPAR binding sites of the promoters. Proteins for EMSA were extracted from HepG2 cell lines. Nuclear and cytoplasmic extracts were prepared based on the methods of Read et al. [55]. Protein concentrations were determined by the BCA method [56]. These extracts were stored at −20 °C until analyzed. Each oligonucleotide duplex of PPAR binding sites was incubated with 10 µg nuclear extracts at room temperature according to the instruction of LightShift™ Chemiluminescent EMSA Kit (Invitrogen), and each unlabeled probe was pre-incubated 10 min prior to the addition of biotin-labeled probe. The reaction was allowed to proceed for 30 min after the addition of biotin-labeled probe at room temperature, and then were detected by electrophoresis on 6% native polyacrylamide gels. Competition analyses were performed by using 100-fold excess of unlabeled oligonucleotide duplex with or without the mutation. All the oligonucleotide sequences of EMSA were listed in Table 1.

### 4.9. Statistical Analysis

Results were presented as mean ± SEM (standard errors of means) in at least three independent biological experiments. Prior to statistical analysis, all data were tested for normality of distribution using the Kolmogornov-Smirnov test. Differences between wild types and drug-treated groups were compared using the Student’s *t* test. Difference was considered significant at *p* < 0.05. All statistical analyses were performed using the SPSS10.0 for Windows (SPSS, Michigan Avenue, Chicago, IL, USA).

## Figures and Tables

**Table 1:** Primers used in the experiments.

Name	Primer	Sequence (5′–3′)
**Primers for promoter construct**		
*CPT Iα1b*	+36R	CCCAAGCTTCTAACAATTCCGATGATGTGG
	−2695F	CGAGCTCAAGCAAGAAGGCAATAGGGT
*CPT Iα2a*	+30R	CCCAAGCTTCGTGCCTTTGATACTAAGTGCG
	−2631F	CGAGCTCGGGCTACCAGTAACTATAAGGG
**Primers for deleting PPAR binding sites of promoters**		
1bMut	- PPAR1F	GCACTTTTCTTTTCCAGAATTTTGTAGTTGTGAGTCA
	- PPAR1R	CTGGAAAAGAAAAGTGCCTTTAATTTCTTGCTC
	- PPAR2F	TGTAGTGGCGACATCTCAGTATCATCTGGGTGG
	- PPAR2R	TGAGATGTCGCCACTACAGGGAGGTGGAAAGGG
2aMut	- PPAR1F	GTTTTACAATTTGTTGGAAATTTTGTTTTGTTTAATG
	- PPAR1R	TCCAACAAATTGTAAAACAAGCATTGCCAACAA
	- PPAR2F	GACTTCGGTAACACTAACAAAACAGTGGGGTAAATC
	- PPAR2R	GTTAGTGTTACCGAAGTCAACATTCTCACATTG
	- PPAR3F	ATGCTCACCGAACAGCTTATGTAAGGCAAGGGA
	- PPAR3R	AAGCTGTTCGGTGAGCATGGAACAGGATTTACC
	- PPAR4F	GGAAGGGGTGATGGAAAAAATCTGTGGTGTCTG
	- PPAR4R	TTTTCCATCACCCCTTCCCTTGCCTTACATAAG
**Oligonucleotide for EMSA assay**		
*CPT Iα1b* - PPAR1	Biotin-probe	Biotin—TAAGCAACTTTGCACTGATTTAC
	Mutative-competitor	TAAGCAACCCCCCACTGATTTAC
*CPT Iα1b* - PPAR2	Biotin-probe	Biotin—ATTTGTTCTTTCCCCCAATGGCC
	Mutative-competitor	ATTTGTTCCCCCCCCCAATGGCC
*CPT Iα2a* - PPAR1	Biotin-probe	Biotin—CGATCAACTATTTCATAGTTGTT
	Mutative-competitor	CGATCAAGGGGTTCATAGTTGTT
*CPT Iα2a* - PPAR2	Biotin-probe	Biotin—AATAATTGTGGGAAAGGTGAAAG
	Mutative-competitor	AATAATTGTGGGGGGGGTGAAAG
*CPT Iα2a* - PPAR3	Biotin-probe	Biotin—TCTTGCTGTGAAATAGGTCAGTT
	Mutative-competitor	TCTTGCTGTGAAGGGGGTCAGTT
*CPT Iα2a* - PPAR3	Biotin-probe	Biotin—GGCTGGGTGGTCTTTTCCCACTT
	Mutative-competitor	GGCCCCCTGGTCTTTTCCCACTT
**Primer for DNase I foot printing assay**		
	M13F	GTAAAACGACGGCCAGT
	M13R-FAM	FAM-CAGGAAACAGCTATGAC

## Supplementary Materials

Supplementary File 1

## Abbreviations

CPT I	carnitine palmitoyltransferase I
FAM	fluorescent 6-carboxy-fluorescein
L-CPT	liver carnitine palmitoyltransferase
LCFA	long-chain fatty acid
M-CPT	muscle carnitine palmitoyltransferase
PCR	polymerase chain reaction
PPAR	peroxisome proliferator-activated receptor
PPRE	peroxisome proliferator-responsive element
RLM-5′RACE	RNA ligase-mediated rapid amplification of 5′ cDNA ends
RXR	retinoid X receptor
TFBS	transcription factor binding site
TR	thyroid hormone receptor
TRE	thyroid hormone response elements
